# Molecular epidemiological study of non-small-cell lung cancer from an environmentally polluted region of Poland

**DOI:** 10.1038/sj.bjc.6690542

**Published:** 1999-07

**Authors:** M Rusin, D Butkiewicz, E Malusecka, A Zborek, J Harasim, K Czyzewski, W P Bennett, P G Shields, A Weston, J A Welsh, S Krzyzowska-Gruca, M Chorazy, C C Harris

**Affiliations:** Department of Tumour Biology, Institute of Oncology, Gliwice, 44-100, Poland; Department of Thoracic Surgery, Silesian Medical Academy, Zabrze, 41-800, Poland; Laboratory of Human Carcinogenesis, National Cancer Institute, National Institutes of Health, Bethesda, MD, 20892-4255, USA; Toxicology and Molecular Biology Branch, National Institute for Occupational Safety and Health, Centers for Disease Control, Morgantown, WA, 26505, USA

**Keywords:** *p53*, WAF1, *GSTM1;*, CYP1A1;, CYP2D6, tobacco smoke

## Abstract

The *p53* mutation spectrum can generate hypotheses linking carcinogen exposure to human cancer. Although it is well-documented that tobacco smoking is a major cause of lung cancer, the contribution of air pollution is less well-established. We determined the molecular and immunohistochemical changes (*p53* gene mutations, p53 protein accumulation and WAF1 protein expression) and genetic polymorphisms of *GSTM1, CYP1A1* and *CYP2D6* genes in a case series of non-small-cell lung cancers from Silesia. This region of southern Poland is highly industrialized with considerable environmental pollution. More than 50% of lung cancers (90/164) contained *p53* mutations and 75% showed the combined alteration of the *p53* gene and protein accumulation. Males occupationally exposed to coal-derived substances showed a relatively high frequency of squamous and large-cell carcinomas, relatively frequent mutations in codon 298 of *p53* and a low frequency of p53 immunohistochemically positive tumours. Codon 298 GAG→ &rarr;TAG mutations have rarely been found in lung cancers in other populations. We found no correlation between WAF1 protein expression and mutations in the *p53* gene or p53 protein accumulation. No statistically significant relationship was found between *p53* mutations and *GSTM1, CYP1A1, CYP2D6* genotypes. Never smokers with lung cancers from Silesia had a higher frequency of G:C→ &rarr;T:A transversions than previously reported of the *p53* mutation spectrum in never smokers (6/15 vs 4/34; *P* = 0.06 by χ^2^). These data are a tentative indication that occupational and environmental exposure to polycyclic aromatic hydrocarbons, such as benzo(a)pyrene, in polluted air contributes to the molecular pathogenesis of lung cancer in never smokers. © 1999 Cancer Research Campaign


*British Journal of Cancer* (1999) **80**(9), 1445–1452
© 1999 Cancer Research Campaign
Article no. bjoc.1999.0542

### Molecular epidemiological study of non-small-cell lung cancer from an environmentally polluted region of Poland

**M Rusin[1], D Butkiewicz[1], E Malusecka[1], A Zborek[1], J Harasim[2], K Czyzewski[2], WP Bennett*, PG Shields[3], A Weston[4], JA Welsh[3], S Krzyzowska-Gruca[1], M Chorazy[1] and CC Harris[3]**

[1]Department of Tumour Biology, Institute of Oncology, 44-100 Gliwice, Poland; [2]Department of Thoracic Surgery, Silesian Medical Academy, 41-800, Zabrze, Poland; [3]Laboratory of Human Carcinogenesis, National Cancer Institute, National Institutes of Health, Bethesda, MD 20892-4255, USA; [4]Toxicology and Molecular Biology Branch, National Institute for Occupational Safety and Health, Centers for Disease Control, Morgantown, WV 26505, USA

**Summary** The *p53* mutation spectrum can generate hypotheses linking carcinogen exposure to human cancer. Although it is well-documented that tobacco smoking is a major cause of lung cancer, the contribution of air pollution is less well-established. We determined the molecular and immunohistochemical changes (*p53* gene mutations, p53 protein accumulation and WAF1 protein expression) and genetic polymorphisms of *GSTM1, CYP1A1* and *CYP2D6* genes in a case series of non-small-cell lung cancers from Silesia. This region of southern Poland is highly industrialized with considerable environmental pollution. More than 50% of lung cancers (90/164) contained *p53* mutations and 75% showed the combined alteration of the *p53* gene and protein accumulation. Males occupationally exposed to coal-derived substances showed a relatively high frequency of squamous and large-cell carcinomas, relatively frequent mutations in codon 298 of *p53* and a low frequency of p53 immunohistochemically positive tumours. Codon 298 GAG→ TAG mutations have rarely been found in lung cancers in other populations. We found no correlation between WAF1 protein expression and mutations in the *p53* gene or p53 protein accumulation. No statistically significant relationship was found between *p53* mutations and *GSTM1, CYP1A1, CYP2D6* genotypes. Never smokers with lung cancers from Silesia had a higher frequency of G:C→ T:A transversions than previously reported of the *p53* mutation spectrum in never smokers (6/15 vs 4/34; $P$ = 0.06 by $\chi^2$). These data are a tentative indication that occupational and environmental exposure to polycyclic aromatic hydrocarbons, such as benzo(a)pyrene, in polluted air contributes to the molecular pathogenesis of lung cancer in never smokers.

**Keywords:** *p53*; WAF1; *GSTM1; CYP1A1; CYP2D6*; tobacco smoke

The *p53* tumour suppressor gene codes for protein that regulates the expression of various genes and affects many cellular functions including: DNA repair, cell cycle checkpoints and apoptosis (reviewed in Harris, 1996; Ko and Prives, 1996; Levine, 1997). The p53 protein activates transcription of the *WAF1/CIP1*, encoding a 21 kDa protein (El-Deiry et al, 1993) associating with the cyclin D1–cyclin- dependent kinase (cdk)4 complex and inhibiting its kinase activity. This in turn prevents the cell from entering the S phase of the cell cycle (Harper et al, 1993; Xiong et al, 1993). Mutations in *p53* lead to the loss of tumour suppressor function and also can result in a gain-of-function phenotype of mutant protein (Gualberto et al, 1998 and refs therein). Missense mutations lead to the abnormal accumulation of p53 protein which can be visualized by immunohistochemical methods (Iggo et al, 1990). Abnormal accumulation of p53 and *p53* mutations are frequent in precancerous lesions (dysplasia) of lung epithelium (Sozzi et al, 1992; Vahakangas et al, 1992; Bennett et al, 1993).

*p53* mutation spectra may give clues to the aetiology of cancer (Hollstein et al, 1991; Harris 1993; Greenblatt et al, 1994; Hussain and Harris, 1998). Thirty per cent of *p53* point mutations in lung cancers from smokers are G:C→ T:A transversions, and most of the G residues are on the DNA non-transcribed strand (Greenblatt et al, 1994). The non-transcribed strand of the *p53* is repaired at a slower rate than the transcribed strand (Evans et al, 1993). G:C→T:A transversions are significantly less frequent in never smokers (Takeshima et al, 1993; Greenblatt et al, 1994; Hainaut et al, 1998: the database for *p53* mutations). These transversions may be attributed to either bulky chemical carcinogens (e.g. benzo[a]pyrene) or, perhaps, oxyradical exposure resulting, for example, from tobacco smoking (Ruggeri et al, 1993; Denissenko et al, 1998).

Tobacco smoking is a predominant cause of human lung cancer (reviewed in IARC Monograph, 1986). The contribution of air pollution to the aetiology of lung cancer is less clear, which may reflect the limited sensitivity of epidemiological studies. The role of occupational and environmental exposure to polluted air is supported by some studies (Miller, 1992; Petersen, 1994; Speizer and Samet, 1994).

Silesia is a highly industrialized and densely populated region in southern Poland with considerable air pollution (Chorazy et al, 1994). Previous studies have shown that non-occupationally exposed Silesian residents have more chromosomal aberrations, sister chromatid exchanges and PAH-DNA adducts than rural controls (Motykiewicz et al, 1992; Perera et al, 1992; Grzybowska et al, 1993; Motykiewicz et al, 1995).

Gene–environment interactions may influence the risk of cancer. Aryl hydrocarbon hydroxylase (AHH) and debrisoquine

*Received 8 June 1998*
*Revised 25 January 1999*
*Accepted 29 January 1999*

*Correspondence to:* CC Harris

*Present address: Division of Human Genetics, City of Hope National Medical Center and Beckman Research Institute, Duarte, CA 91010-3000, USA.

hydroxylase, encoded by *CYP1A1* and *CYP2D6* genes respectively, have been studied extensively in lung cancer patients with varying results. Initial *CYP2D6* phenotyping studies suggested overrepresentation of the dominant EM (extensive metabolizer) phenotype in lung cancer patients when compared to patients with chronic obstructive pulmonary disease (Ayesh et al, 1984; Caporaso et al, 1989). Subsequent genotyping reports have not confirmed these findings (Sugimura et al, 1990; Tefre et al, 1994; Stucker et al, 1995). Caporaso et al (1995) discussed the possible explanation of these apparent paradoxical findings. For *CYP1A1*, the polymorphic minor allele codes for valine in exon 7 (codon 462) instead of isoleucine. A significant preponderance of the susceptible *Val/Val* genotype among individuals with lung cancer has been reported in a Japanese population (Hayashi et al, 1992). The glutathione-S-transferase (*GSTM1*) gene encodes glutathione S-transferase class μ that catalyses conjugation reactions of glutathione with electrophiles (e.g. activated PAHs). Some reports have observed that lack of GSTM1 activity due to the homozygous deletion confers increased lung cancer risk (Hirvonen et al, 1993; Alexandrie et al, 1994; Kihara et al, 1994; reviewed by Rebbeck, 1997). This increased lung cancer risk is compounded for carriers of *CYP1A1* susceptibility genotypes (Hayashi et al, 1992; Nakachi et al, 1993).

The specific aim of our investigation was to determine molecular and immunohistochemical changes (*p53* gene mutations, p53 protein accumulation and WAF1 protein expression) in a case series of non-small-cell lung cancer (NSCLC) samples from a heavily polluted area of Poland. We investigated the hypothesis that the *p53* mutational spectrum in the Silesian residents reflects the mutagenic activity of air pollution. Our analyses also examined possible relationship between the mutational spectrum of *p53* and selected functional genetic polymorphisms involved in carcinogen metabolism.

### MATERIALS AND METHODS

### Sample collection

The cases of NSCLC were resected at the Department of Thoracic Surgery, Silesian Medical Academy between 1991 and 1995. The patients were interviewed at the hospital by either a physician or a trained nurse. Data on demographics, medical history, family history of cancer, occupational exposure and smoking habits were collected by questionnaire. Tumour and non-tumour samples were either frozen on dry ice (all non-tumour lung and 45 tumour samples) or fixed (see below) and paraffin-embedded (119 tumour samples). Forty-five tumour samples were not available for paraffin embedding. DNA from fresh tissues was extracted, after crushing in liquid nitrogen, using standard procedures (Sambrook et al, 1989). Cancer cells were not microdissected from tumour samples. DNA from dewaxed paraffin sections of tumour tissues was extracted by sodium dodecyl sulphate (SDS)–proteinase K treatment (0.5 mg $ml^{-1}$ in 1% SDS) at 55°C for 18–24 h followed by phenol–chloroform extraction and ethanol precipitation with glycogen as a carrier.

### Immunohistochemistry

Immediately after the surgical removal, the tissues were fixed for 24 h in either ice-cold 10% formalin in phosphate-buffered saline (PBS), or in methacarn (methanol:chloroform:acetic acid 6:3:1) and embedded in paraffin. A portion of each specimen also was fixed by 4% paraformaldehyde in PBS. Immunohistochemical staining was performed on dewaxed 7-μm sections. Formalin-fixed tissues sections were heated $2 \times 5$ min in a microwave oven in 0.01 M citric buffer, pH 6.0. The p53 protein was detected using either the polyclonal antibody CM1 (Signet Laboratories) or one of the following monoclonal antibodies: Ab 1801 (Ab 2, Oncogene Sciences, Gaithersburg, MD, USA), DO-1 (Ab 6, Oncogene Sciences) recognizing epitopes on the amino-terminus of the p53 protein chain (amino acids 40–65 and 37–45 respectively), HR231 (a gift of Dr T Soussi) that reacts with amino acids 351–393 on the carboxy-terminus of the p53 protein (Legros et al, 1993). All used antibodies recognize both the wild-type and mutant p53. The four anti-p53 antibodies yielded a very similar staining pattern, but the intensity of staining varied slightly. There was no instance of a positive result with one antibody contradicted by lack of staining with another.

For detection of the $p21^{WAF1/CIP1}$ protein, sections of tissue fixed in formalin and exposed to microwave antigen retrieval were used. The sections were incubated overnight at 4°C with the primary antibody (mouse monoclonal antibody Ab-1 from Oncogene Sciences). Indirect immunoperoxidase staining with avidin–biotin or streptavidin peroxidase complexes and 3,3′-diaminobenzidine as a substrate was applied for visualization of the antigens. The sections were counterstained with Mayer's haematoxylin.

p53 and $p21^{Waf1}$ immunohistochemical analysis of the intensity of positive staining was scored as follows: + 2–10% of cells distinctly positive or more cells weakly stained, ++ 10–50% distinctly positive or more cells weakly stained, +++ more than 50% of cells strongly stained.

### Mutational analysis of the *p53* gene

Exons 5 to 8 of *p53* were amplified by polymerase chain reaction (PCR) with intronic primers (Lehman et al, 1991) and sequenced with Sequenase Version 2.0 DNA sequencing kit (USB-Amersham, Cleveland, OH, USA). When PCR amplification was strong, the PCR product was sequenced with Sequenase PCR product sequencing kit (USB-Amersham). A DNA fragment with mutation was reamplified and resequenced to confirm that the mutation was not introduced by an error of the thermostable polymerase used for PCR. Germline mutations were excluded by PCR amplification and sequencing of relevant exons from non-tumour lung DNA.

### Genotyping of *GSTM1*, *CYP2D6* and *CYP1A1*

The *CYP1A1* exon 7 and *GSTM1* polymorphisms were determined as described previously (Shields et al, 1993). In a multiplex PCR reaction, a *CYP1A1* fragment served as an internal control for the detection of the *GSTM1* gene deletion. The *CYP1A1* fragment was analysed by *Nco*I restriction enzyme digestion. The *CYP2D6-A* allelic variant was detected using allele-specific double-step PCR according to Heim and Meyer (1990). The *CYP2D6-B* allele was identified using PCR and *Bst*NI digestion as previously described by Kato et al (1995).

### Statistical analyses

Descriptive statistics and standard measures of association (e.g. cross-tabulations using $\chi^2$ test for association) were used for data

 © 1999 Cancer Research Campaign

**Table 1** Frequency of *p53* mutations in non-small-cell lung cancers from Silesia, Poland

| | All mutations | SQ | AD | LG | Smokers | Never smokers | Coal-exposed | Coal non-exposed |
|---|---|---|---|---|---|---|---|---|
| G:C→T:A | 31 | 20 | 55 | 50 | 29 | 39 | 37 | 24 |
| G:C→A:T | 29 | 33 | 30 | 0 | 31 | 20 | 20 | 34 |
| G:C→C:G | 9 | 12 | 0 | 10 | 9.5 | 7 | 8 | 12 |
| A:T→T:A | 4.5 | 5 | 0 | 10 | 4 | 7 | 2.5 | 5 |
| A:T→C:G | 5.5 | 5 | 5 | 10 | 5 | 7 | 5 | 10 |
| A:T→G:C | 9 | 10 | 10 | 0 | 9.5 | 7 | 12.5 | 5 |
| del/ins | 12 | 15 | 0 | 20 | 12 | 13 | 15 | 10 |
| Number of mutations | *n* = 90 | *n* = 59 | *n* = 20 | *n* = 10 | *n* = 75 | *n* = 15 | *n* = 40 | *n* = 41 |

Except for the bottom row the numbers are per cents. Two mutations are not included because they represent either tandem or composite mutation. One mutation is not included in the histological column because the tumour was of mixed type. Coal-exposed and non-exposed patients were males. Using mutational spectra comparison software, the only significant differences were found between spectra in SQ vs AD ($P = 0.042$; Monte Carlo analysis) and between AD vs LG ($P = 0.029$; Monte Carlo analysis). Moreover, the difference of G:C→T:A frequencies in SQ vs AD was also significant ($P = 0.008$; $\chi^2$).

**Table 2** Tumour types, p53 immunohistochemical staining and *p53* mutations in coal-exposed and non-exposed NSCLC cases (males only)

| | Coal-exposed | Non-exposed | Significance |
|---|---|---|---|
| SQ + LG | 59 (84%) | 46 (68%) | |
| AD | 11 (16%) | 22 (32%) | $P = 0.04$ $\chi^2$ |
| p53 IHC-positive | 24 (50%) | 40 (77%) | |
| p53 IHC-negative | 24 (50%) | 12 (23%) | $P = 0.01$ $\chi^2$ |
| *p53* mutations | 39 (56%)[a] | 41 (60%) | |
| No *p53* mutation detected | 31 (44%) | 27 (40%) | $P = 0.71$ $\chi^2$ |
| *p53* missense mutation | 26 (67%) | 30 (75%) | |
| Other *p53* mutations[b] | 13 (33%) | 10 (25%)[c] | $P = 0.57$ $\chi^2$ |
| Non-codon 298 mutation | 35 (90%) | 41 (100%) | |
| Codon 298: GAG→TAG | 4 (10%) | 0 | $P = 0.05$ Fisher's |

[a]One exposed patient had two mutations; [b]Non-sense mutations, deletions and frameshifts; [c]One silent mutation was not included. SQ, squamous cell carcinoma; AD, adenocarcinoma; IHC, immunohistochemistry.

**Table 3** Gender, smoking status and *GSTM1*, *CYP1A1* and *CYP2D6* genotypes

| | ***GSTM1–*** | | ***CYP1A1*** | | ***CYP2D6*** | | |
|---|---|---|---|---|---|---|---|
| | Null | Positive | Ile/Ile | Ile/Val | BB or AB | WT + A or B | WT/WT |
| Females | 13 (62%) | 8 (38%) | 17 (81%) | 4 (19%) | 2 (9.5%) | 8 (38%) | 11 (52.5%) |
| Males | 70 (52%) | 65 (48%) 1* | 115 (85%) | 20 (15%) 2* | 7 (5%) | 51 (38%) | 77 (57%) 3* |
| Smokers | 69 (52%) | 64 (48%) | 114 (86%) | 19 (14%) | 8 (6%) | 50 (38%) | 75 (56%) |
| Never smokers | 14 (61%) | 9 (39%) 4* | 18 (78%) | 5 (22%) 5* | 1 (4%) | 9 (39%) | 13 (57%) 6* |

1* $P = 0.53$ $\chi^2$; 2* $P = 0.86$ Yates corrected $\chi^2$; 3* $P = 0.77$ Yates corrected $\chi^2$ (*BB* or *AB* vs others); 4* $P = 0.57$ $\chi^2$; 5* $P = 0.54$ $\chi^2$; 6* $P = 0.87$ Yates corrected $\chi^2$ (*BB* or *AB* vs others).

exploration and to test the significance. These analyses were performed using the Statistica Version 5.0 program for PCs (StatSoft, Inc., Tulsa, OK, USA) or Statistical Analysis System (Cary, NC, USA). Mutational spectra of the p53 were compared using a computer program developed by Cariello et al (1994). It can be obtained at *http://sunsite.unc.edu/dnam/mainpage.html*. We refer to it as Monte Carlo analysis. The differences were considered statistically significant if the *P*-value was 0.05 or less in two-tailed tests.

### RESULTS

Exons 5–8 of *p53* were sequenced directly after PCR amplification from genomic DNA of 164 patients without pre-selection of samples. Ninety-two somatic mutations were found in 90 patients (55%). In two patients, we found two *p53* mutations. In sample no. 288, which stained positively for the p53 protein, one base pair deletion in exon 5 (codon 159) and a mis-sense mutation in exon 8 (codon 275) were found. In sample no. 217, which stained positively

*© 1999 Cancer Research Campaign* 

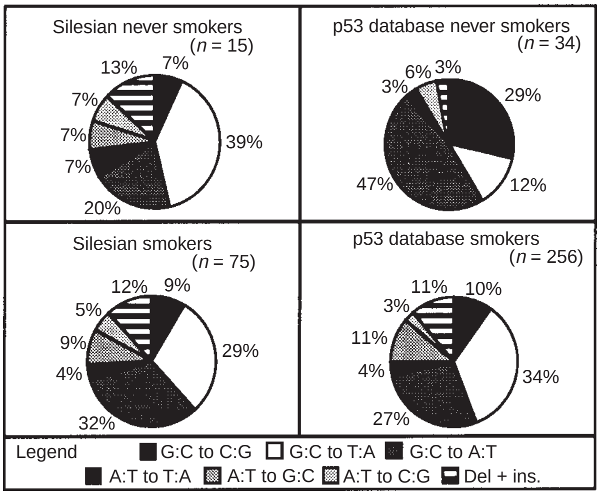

**Figure 1** Mutation spectra of *p53* in NSCLC from Silesian patients and from the database of *p53* mutations (Hainaut et al, 1998). Note that the spectra of Silesian smokers, never smokers and smokers from the database are almost identical (Silesia smokers vs database smokers, $P = 0.89$; Monte Carlo analysis), whereas the spectrum of *p53* mutations in never smokers from the database is significantly different from Silesian never smokers ($P = 0.03$; Monte Carlo analysis)

for p53 protein, a non-sense mutation in exon 8 (codon 298) and a missense mutation in exon 7 (codon 246) were detected.

The frequency of *p53* mutations in large-cell carcinomas (LG), squamous cell carcinomas (SQ) and adenocarcinomas (AD) was: 83% (10/12), 56% (60/107) and 43% (19/44) respectively (one tumour was diagnosed as of mixed histology).

A statistically significant difference of *p53* mutation frequency was found between AD and LG ($P = 0.02$, Fisher's exact test), the other differences were not statistically significant. No statistically significant association was detected between the presence of *p53* gene mutations and gender, T stage (characterizing primary tumour size), N stage (characterizing presence of cancer invasion to local lymph nodes) or general stage (I, II or III) of lung cancer (combining the data on: primary tumour size, spread of cancer tissue to local lymph nodes, presence on distant metastases).

Five mutational hotspots were detected (with at least four mutations in each) at codons: 155 ($n = 4$), 158 ($n = 4$), 248 ($n = 7$), 273 ($n = 5$) and 298 ($n = 5$). Mutational spectra of the *p53* gene in the selected groups of patients and the spectrum of all mutations are shown in Table 1. It was found that the spectra in smokers and never smokers do not differ significantly, although G:C→T:A transversions were more frequent in never smokers (39%) compared with smokers (29%; Table 1). We did not find a dose–response relation between the number of smoked cigarettes (pack-years) and either the frequency of mutations or the frequency of G:C→T:A transversions (data not shown).

In our series of patients, 70 males were occupationally exposed to either coal dust or coal processing substances (54 coal miners, but also foundry workers and others). The percentage of histological types in coal-exposed versus non-exposed individuals (males only) is shown in Table 2. The observed differences are statistically significant ($P = 0.04$, $\chi^2$ test). Four non-sense mutations in codon 298 (GAG→TAG; Glu→STOP) were found among 39 coal-exposed males with mutated *p53*, whereas no non-exposed male patient out of 41 with mutated *p53* showed this mutation ($P = 0.05$ by Fisher's exact test). The overall spectra in coal-exposed and in non-exposed male patients did not differ (Table 1). We observed that tumours of non-coal-exposed patients tended to stain positively for p53 (40/52, 77%) when compared to coal-exposed patients (24/48, 50%); ($P = 0.01$, $\chi^2$, Table 2). No significant association between the exposure to coal and the presence of *p53* gene mutations (Table 2) was detected, although, consistent with immunohistochemical data, males exposed to coal had more mutations (frameshifts, splice site mutations, non-sense mutations) coding for truncated, rapidly degraded p53 molecules, than non-exposed males (33% and 25% respectively; $P = 0.57$ by $\chi^2$, Table 2).

G:C→T:A transversions were more frequent in males (26/82, 32%) than in females (2/8, 25%), but the difference was not statistically significant. The frequency of all *p53* mutations was lower in females (8/21, 38%) compared to males (82/143, 57%; $P = 0.16$ by $\chi^2$). The gender difference in the frequency of *p53* mutations (although not significant) is likely related to lower frequencies of *p53* mutations in AD which have, in our sample set, a higher prevalence in females (10/21; 48%) compared to males (34/143; 24%; $P = 0.04$ by $\chi^2$).

In our case series AD were more frequent in never smokers (9/23; 39%) than in smokers (35/140; 25%; $P = 0.25$ by $\chi^2$). But in never smokers with a mutated *p53* gene (15 individuals) AD constitute only 26% of cases (four in 15) and only one of the AD had G:C→T:A transversion. Thus, most of G:C→T:A transversions frequently observed in never smokers (39%) are found in SQ and LG.

G:C→T:A transversions showed a strong strand bias: 96% of G nucleotides were located on the DNA coding (non-transcribed) strand and only 4% on the non-coding (transcribed) strand. The bias was stronger in smokers (100%) than in never smokers (86%). In contrast, the DNA strand bias was not observed for G:C→A:T transitions (54% of G nucleotides on coding strand, 46% on non-coding strand).

The *GSTM1*, *CYP1A1* (exon 7 polymorphism) and *CYP2D6* (alleles *A* and *B*) genotypes were determined in 156 NSCLC patients (Table 3). Normal tissue from eight cases was not available. Eighty-three patients were found to be *GSTM1-null* (53%) and 24 patients were heterozygous for exon 7 polymorphism of *CYP1A1* gene (15%). We did not find any patient who was homozygous for the minor *Val* allele of *CYP1A1* (allele frequency 0.08). For *CYP2D6*, we found 88 (56%) patients who had neither *A* nor *B* alleles (predicted phenotype EM), 59 (38%) patients who were heterozygous for one of the mutant alleles *A* or *B* (predicted phenotype IM – intermediate metabolizers), and nine (6%) patients who carried either two *B* alleles or *A* and *B* alleles t (predicted phenotype PM – poor metabolizers).

No statistically significant gender differences were found of genotype frequencies (Table 3) although the *GSTM1-null* genotype was more frequent in females. The genotype frequencies in smokers and in never smokers are shown in Table 3. Analysis of genotype frequencies by smoking history showed that frequencies of 'at risk' genotypes of *GSTM1* (*null*) and *CYP1A1* (*Ile/Val*) were higher in never smokers than in smokers, but the differences were not statistically significant (Table 3). The relation between genotypes and *p53* mutations and p53 protein overexpression are shown in Table 4. No statistically significant associations between the variables were observed.

We did not observe any statistically significant association between combined *GSTM1* and *CYP1A1* polymorphisms (*GSTM1-positive/CYP1A1 Ile/Val, GSTM1-positive/CYP1A1 Ile/Ile, GSTM1-null/CYP1A1 Ile/Val, GSTM1-null/CYP1A1 Ile/Ile*) and the muta-

 *© 1999 Cancer Research Campaign*

**Table 4** *GSTM1*, *CYP1A1* and *CYP2D6* genotypes and *p53* gene and protein

| Genotype | *p53* seq | | p53 IHC | | p53 seq + IHC | |
|---|---|---|---|---|---|---|
| *GSTM1* | WT | MUT | Negative | Positive | Not altered | Altered |
| *Null* | 39 (47%) | 44 (53%) | 19 (32%) | 41 (68%) | 13 (16%) | 70 (84%) |
| *Positive* | 30 (41%) | 43 (59%) 1* | 22 (42%) | 30 (58%) 2* | 14 (19%) | 59 (81%) 3* |
| *CYP1A1* | *p53* seq | | p53 IHC | | p53 seq + IHC | |
| *Ile/Ile* | 57 (43%) | 75 (57%) | 36 (38%) | 60 (62%) | 25 (19%) | 107 (81%) |
| *Ile/Val* | 12 (50%) | 12 (50%) 4* | 5 (31%) | 11 (69%) 5* | 2 (8%) | 22 (92%) 6* |
| *CYP2D6* | *p53* seq | | p53 IHC | | p53 seq + IHC | |
| *BB* or *AB* | 3 (33%) | 6 (67%) | 3 (50%) | 3 (50%) | 2 (22%) | 7 (78%) |
| *WT/B* or *WT/A* or *WT/WT* | 66 (45%) | 81 (55%) 7* | 38 (36%) | 68 (64%) 8* | 25 (17%) | 122 (83%) 9* |

1* $P = 0.56$ $\chi^2$; 2* $P = 0.33$ $\chi^2$; 3* $P = 0.71$ $\chi^2$; 4* $P = 0.69$ $\chi^2$; 5* $P = 0.84$ $\chi^2$; 6* $P = 0.33$ Yates corrected $\chi^2$; 7* $P = 0.74$ Yates corrected $\chi^2$; 8* $P = 0.79$ Yates corrected $\chi^2$; 9* $P = 0.96$ Yates corrected $\chi^2$.

tions in *p53* in a group of 133 smokers for whom the genotyping data were available. We also compared the mutation spectra of the *p53* gene among *GSTM1-positive* and *GSTM1-null* smokers. The *p53* mutational spectra in neither group showed statistically significant differences although the deletions/insertions were more frequent in *GSTM1-positive* individuals (7/40; 18%) than in *GSTM1-null* ones (2/34; 6%; $P = 0.17$ Fisher's exact test). In never smokers we found that almost all (12/13; 92%) *GSTM1-null* individuals stained positive for the p53 protein whereas only 38% (3/8) *GSTM1-positive* individuals stained positive for the p53 protein ($P = 0.014$ by Fisher's exact test). A similar relation was observed in never smokers for the mutations of the *p53* gene: 79% (11/14) *GSTM-null* individuals had a mutated p53 gene, whereas only 44% (4/9) *GSTM1-positive* individuals had mutations in *p53*; however, this difference was not statistically significant ($P = 0.18$; Fisher's exact test).

The accumulation of p53 protein determined by immunohistochemical methods was examined in 119 cases. Immunostaining for p53 was most prominent in the formalin-fixed tissue after microwave treatment, distinct in methacarn-fixed tissue and often either a low or false-negative in formalin-fixed material without antigen retrieval. The p53 immunostaining was found almost exclusively in nuclei of cancer cells. In some cases, single positive cell nuclei were visible in bronchial epithelium or among cells of lymph nodules. Distinct cytoplasmic localization was found in positive cases in some mitotic cancer cells. Accumulation of p53 was detected in 75 (63%) out of a total of 119 tumours examined for p53 protein expression.

All 119 cases examined for p53 protein staining were also analysed by the sequencing of exons 5–8 of *p53*. There were no missense mutations among p53 immunohistochemically negative cases. Thirty of the 119 cases (25%) showed neither mutation of the *p53* gene nor accumulation of the p53 protein, whereas 89 cases (75%) showed either mutations of the *p53* gene (14/89) or an accumulation of p53 protein (21/89) or both (54/89). Statistical analyses (Student's *t*-test) showed that the mutation of the *p53* gene is significantly associated with strong immunohistochemical staining of the p53 protein ($P = 0.001$). We found no association between the stage of the disease and the immunohistochemical staining of the p53 protein.

The immunohistochemical analyses of the $p21^{WAF1/CIP1}$ antigen were studied in 119 NSCLC cases immunostained also for p53 protein. Of three fixatives used, both PAF and formalin yielded positive immunostaining of $p21^{WAF1/CIP1}$ after antigen retrieval, while methacarn proved to be unsuitable for the purpose. The $p21^{WAF1/CIP1}$ staining was found to be localized in tumour cell nuclei. In some cases, single nuclei of the bronchial epithelium or cells within lymph nodules were also positive. A positive reaction for $p21^{WAF1/CIP1}$ was present in 82 cases (69%). The majority of $p21^{WAF1/CIP1}$-positive cases also were reactive with anti-p53 antibodies (56/82; 68%). Samples showing a strong immunohistochemical reaction for p21 protein (++) more frequently showed strong (++ or +++) (19/25; 76%) rather than a weak or negative (6/25; 24%) staining for p53 protein. Among 37 cases negative for $p21^{WAF1/CIP1}$ staining, only 19 (51%) showed a positive immunostaining for p53.

In some tumour foci of well-differentiated squamous cell carcinomas, the immunoreaction for p53 was observed in basal cell layers, whereas $p21^{WAF1/CIP1}$-positive cells were localized in layers of differentiating cells, sometimes displaying the features of keratinization. In other cases, the positive cells were randomly distributed in the cancer tissue. Co-localization of both p53 and $p21^{WAF1}$ antigens in the same cell nuclei was also observed.

No association was found between $p21^{WAF1/CIP1}$ IHC staining and any of the following: gender, smoking status, histology, occupational exposure, the stage of the disease or the mutation of the *p53* gene (data not shown). When immunohistochemical staining for p21 and p53 was combined, no association between combined staining (both negative, either positive, both positive) and gender, histology or disease stage was found (data not shown).

### DISCUSSION

The DNA sequence was determined for exons 5–8 of *p53* of 164 primary NSCLC from Silesia, a heavily polluted region of Poland. Mutations of *p53* were found in tumours of 90 patients (55%). The most frequent mutation type was G:C→T:A transversions (31%), which is in agreement with the data of other authors (Figure 1) (Greenblatt et al, 1994; Hainaut et al, 1998: the database of *p53*

© 1999 Cancer Research Campaign 

mutations). Surprisingly, G:C→T:A transversions were more frequent, although not significantly, in Silesian never smokers than in smokers (39% and 29% respectively, Table 1 and Figure 1). This was in contrast with *p53* mutational spectrum in never smokers from other populations (Figure 1) which showed a predominance of G:C→A:T transitions (47%). Unfortunately, the lung cancer incidence in Silesian never smokers is not known. Our data are a tentative indication that occupational and environmental exposure to polluted air contributes to the molecular pathogenesis of lung cancer in never smokers. To test this hypothesis further, the *p53* mutation data from never smokers living in less polluted areas are needed.

In contrast to other reports (Takeshima et al, 1993; Wang et al, 1995; Kondo et al, 1996), we did not find a dose–response relation between the number of smoked cigarettes (pack-years) and either the frequency of all *p53* mutations or the frequency of *p53* G:C→T:A transversions, which may be explained by the confounding effect of environmental air pollutants. Also it is of interest that 100% of the G:C→T:A transversions in smokers (22 mutations) showed DNA strand bias for the DNA coding strand, which was higher than in other populations studied (reviewed by Greenblatt et al, 1994). The similarity of the mutational spectrum in smokers from our case series and spectra reported by others from Caucasian and Oriental populations may result from a considerable influence that the carcinogens in tobacco smoke have on the mutational processes in target lung cells apart from either the differences in local environment or the differences in genetic background of populations. The spectrum of mutations in *p53* in never smokers may reflect also differences in either local environment or in the genetic makeup of populations. Future molecular epidemiological studies on *p53* mutations from never smoking lung cancer patients of various ethnic, environmental and occupational backgrounds will clarify these issues.

We detected five *p53* mutational hotspots. The most intriguing one is codon 298. Four of its G to T transversions are found in coal-exposed males (all were coal miners) and only one in a non-miner. Two of the miners were never smokers. This mutation is very rare in lung cancers from other geographical regions of the world. The frequency difference is significant for all *p53* mutations in NSCLC in the database (5/92 vs 6/495; $P = 0.03$; Yates corrected $\chi^2$; Hainaut et al, 1998). These data generate the hypothesis that the codon 298 GAG to TAG mutation in coal miners is induced by a carcinogen to which the miners are exposed. According to our knowledge this is the first report of the spectrum of *p53* mutations in lung cancers from coal miners. We also noted that males exposed to coal dust and coal-processing substances differed significantly from non-exposed males in frequency of histological types. Adenocarcinomas were much less frequent in exposed than in non-exposed males and non-adenocarcinomas were associated with coal exposure. It is of further interest that tumours in males occupationally non-exposed to coal stained more frequently for the p53 protein than tumours of occupationally-exposed males ($P = 0.01$ by $\chi^2$). This, in part, could be explained by a higher frequency of loss-of-function mutations in coal-exposed males (33%) compared with non-exposed ones (25%; Table 2). The apparent discrepancy between the p53 immunohistochemistry status and *p53* sequencing data in Table 2, can result from the fact that small deletions, insertions and non-sense mutations generally lead to no detectable p53 protein and, therefore, negative p53 immunohistochemistry staining. Conversely, positive p53 immunohistochemistry staining can result from other mechanisms than *p53* mutations that are not understood at present.

The epidemiological data indicate that lung cancer mortality is not elevated in Polish coal miners (Starzynski et al, 1996). This is consistent with the results of studies performed on coal miners from other countries, e.g. UK, USA, The Netherlands (Miller and Jacobsen, 1985; Meijers et al, 1991; Kuempel et al, 1995). We do not suggest that there is a substantial lung cancer risk in coal miners. Most of the coal miners from our patient series were smokers. It is conceivable that some carcinogens in coal mines with concurrent tobacco smoking do not substantially increase lung cancer risk but induce a different ‘pathway’ of cancer formation, hence, for example, an elevated frequency of non-adenocarcinomas and p53 immunohistochemistry-negative tumours in coal exposed patients (Table 2). The similar analyses performed on coal miners from other countries would be of great value.

Significant differences in the *p53* mutational spectra between adenocarcinomas and squamous cell carcinomas and between adenocarcinomas and large cell carcinomas were detected in our case series. The most striking difference in mutational spectra between adenocarcinomas and squamous cell carcinomas was the frequency of G:C→T:A transversions, which are much more frequent in adenocarcinomas (55%) than in squamous cell carcinomas (20%; $P = 0.008$; $\chi^2$; Table 1). When only G:C→T:A transversions showing DNA strand bias (G base located on DNA coding strand) were considered, the difference was even more obvious (55% vs 19%).

The higher susceptibility to lung cancer was associated with the exon 7 polymorphism of *CYP1A1* gene in Japanese population. The frequency of *Val* allele in Japanese lung cancer patients is 0.26 (Nakachi et al, 1993). It was also shown that the *p53* gene is more frequently mutated in patients with the *CYP1A1 Val/Val* genotype (Kawajirii et al, 1996; Przygodzki et al, 1998). We have not detected the similar relationship in our case series (not shown). However, only 15% of the patients were heterozygous for the *Val* allele so that our study does not have sufficient statistical power to adequately test the hypothesis proposed by Kawajiri et al (1996).

*GSTM1-null* heavy smokers have a higher risk of transversion mutations in the *p53* gene (Ryberg et al, 1994), although it was not observed by other investigators (Kawajirii et al, 1996). In the Silesian cases, the spectrum of *p53* mutations is similar among smokers who are *GSTM1* wild-type and null. Moreover, in contrast with the report of Ryberg et al (1994) *GSTM1*-positive patients from our case series tend to have a higher *p53* mutation frequency than *GSTM1*-deficient individuals (Table 4). Taken together, our data indicate that the influence of GSTM1 on *p53* mutagenesis is small in the Silesian cases.

The role of *CYP2D6* in modulating lung cancer susceptibility is controversial. So far, two tobacco smoke components: 4-(methylnitrosamino)-1-(3-pyridyl)-1-butanone (NNK) and nicotine, have been proposed as substrates for CYP2D6 (Crespi et al, 1991; Cholerton et al, 1994). The frequency of predicted phenotypes for our group of patients is almost identical to the frequency of phenotypes determined by pharmacogenetic methods in a healthy Polish population (EM: 58% by genotyping vs 57% by metabolite measurements, IM 37% vs 37%, PM 5% vs 6%) (Kunicki et al, 1995). Considering the *CYP2D6* genotype (presence of alleles *A* and *B*), we classified the patients by the two predicted phenotypes (EM or IM and PM). Since we detected only nine patients with predicted PM phenotype, it was not possible to reasonably compare mutational spectra between *CYP2D6* proficient and deficient individuals.

Our study demonstrated that the use of the anti-p21$^{WAF1/CIP1}$ anti-

 *© 1999 Cancer Research Campaign*

body stained nuclei of tumour cells selectively, leaving the normal lung tissue unstained. In our material, the *p21*$^{WAF1/CIP1}$ product occurred independently of the status of p53 protein or gene. Study performed on a different NSCLC case series from the USA yielded similar results (Bennett et al, 1998). In conclusion, we observed that more than 50% of NSCLC patients had mutations within *p53* in DNA isolated from tumour tissues. In contrast with other studies (reviewed in Greenblatt et al, 1994; Hainaut et al, 1998; Hussain and Harris, 1998), the never smokers had a high frequency of G:C→T:A transversions. We observed that males exposed occupationally to coal dust and coal processing substances (mostly coal miners) constitute a group of patients that can be characterized by a relatively high frequency of squamous and large-cell carcinomas, relatively frequent nonsense mutations in codon 298 of *p53*, and a relatively low frequency of p53 immunohistochemistry-positive tumours. These data are a tentative indication that occupational and environmental exposure to polluted air contributes to the molecular pathogenesis of lung cancer in Silesian residents.

### ACKNOWLEDGEMENTS

This work was supported in part by grants from the State Committee for Scientific Research (KBN) # 6P20706405p03, # 6P20706405p02 for the Department of Tumour Biology and profited also from Polish-American MSC Fund II grant # MZ/HHS-93-133. MC thanks the Fogarty International Center, NIH, MD, USA for fellowship, 5F05. TW04836-03. We would like to thank Ms A Kyrcz, Ms I Matuszczyk, Ms H Paterak, Ms I Peick, Ms H Waniek and Mr A Samojedny for their excellent technical assistance.

The editorial assistance of Ms Dorothea Dudek is also appreciated.

### REFERENCES

Alexandrie AK, Ingelman Sundberg M, Seidegard J, Tornling G and Rannung A (1994) Genetic susceptibility to lung cancer with special emphasis on CYP1A1 and GSTM1: a study on host factors in relation to age at onset, gender and histological cancer types. *Carcinogenesis* **15**: 1785–1790

Ayesh R, Idle JR, Ritchie JC, Crothers MJ and Hetzel MR (1984) Metabolic oxidation phenotypes as markers for susceptibility to lung cancer. *Nature* **312**: 169–170

Bennett WP, Colby TV, Travis WD, Borkowski A, Jones RT, Lane DP, Metcalf RA, Samet JM, Takeshima Y, Gu JR, Vahakangas KH, Soini Y, Paakko P, Welsh JA, Trump BF and Harris CC (1993) p53 protein accumulates frequently in early bronchial neoplasia. *Cancer Res* **53**: 4817–4822

Bennett WP, El-Deiry WS, Guinee DG, Freedman AN, Rush WL, Caporaso NE, Welsh JA, Jones RT, Borkowski A, Travis WD, Fleming MV, Trastek V, Pairolero PC, Tazelaar HD, Midthun D, Jett JR, Liotta LA and Harris CC (1998) p21$^{waf1/cip1}$ and transforming growth factor β1 protein expression correlate with survival in non-small cell lung cancer. *Clin Cancer Res* **4**: 1499–1506

Caporaso N, Hayes RB, Dosemeci M, Hoover R, Ayesh R, Hetzel M and Idle J (1989) Lung cancer risk, occupational exposure, and the debrisoquine metabolic phenotype. *Cancer Res* **49**: 3675–3679

Caporaso N, DeBaun MR and Rothman N (1995) Lung cancer and CYP2D6 (the debrisoquine polymorphism): sources of heterogeneity in the proposed association. *Pharmacogenetics* **5**: 129–134

Cariello NF, Piegorsch WW, Adams WT and Skopek TR (1994) Computer program for the analysis of mutational spectra: application to p53 mutations. *Carcinogenesis* **15**: 2281–2285

Cholerton S, Arpanahi A, McCracken N, Boustead C, Taber H, Johnstone E, Leathart J, Daly AK and Idle JR (1994) Poor metabolisers of nicotine and CYP2D6 polymorphism. *Lancet* **343**: 62–63

Chorazy M, Szeliga J, Strozyk M and Cimander B (1994) Ambient air pollutants in Upper Silesia: partial chemical composition and biological activity. *Environ Health Perspect* **102**: 61–66

Crespi CL, Penman BW, Gelboin HV and Gonzales FJ (1991) A tobacco smoke-derived nitrosoamine, 4-(methylnitrosamino)-1-(3-pyridyl)-1-butanone, is activated by multiple human cytochrome P450s including the polymorphic human cytochrome P4502D6. *Carcinogenesis* **12**: 1197–1201

Denissenko MF, Pao A, Pfeifer GP and Tang M (1998) Slow repair of bulky DNA adducts along the nontranscribed strand of the human p53 gene may explain the strand bias of transversion mutations in cancers. *Oncogene* **16**: 1241–1247

El-Deiry WS, Tokino T, Velculescu VE, Levy DB, Parsons R, Trent JM, Lin D, Mercer WE, Kinzler KW and Vogelstein B (1993) WAF1, a potential mediator of p53 tumor suppression. *Cell* **75**: 817–825

Evans MK, Taffe BG, Harris CC and Bohr VA (1993) DNA strand bias in the repair of the p53 gene in normal human and xeroderma pigmentosum group C fibroblasts. *Cancer Res* **53**: 5377–5381

Greenblatt MS, Bennett WP, Hollstein M and Harris CC (1994) Mutations in the p53 tumor suppressor gene: clues to cancer etiology and molecular pathogenesis. *Cancer Res* **54**: 4855–4878

Grzybowska E, Hemminki K and Chorazy M (1993) Seasonal variation in level of DNA adducts and X-spots in human populations living in different parts of Poland. *Environ Health Perspect* **99**: 77–81

Gualberto A, Aldape K, Kozakiewicz K and Tlsty TD (1998) An oncogenic form of p53 confers a dominant, gain-of-function phenotype that disrupts spindle checkpoint control. *Proc Natl Acad Sci USA* **95**: 5166–5171

Haupt Y, Maya R, Kazaz A and Oren M (1997) Mdm2 promotes the rapid degradation of p53. *Nature* **387**: 296–299

Hainaut P, Hernandez T, Robinson A, Rodriguez-Tome P, Flores T, Hollstein M, Harris CC and Montesano R (1998) IARC Database of p53 gene mutations in human tumors and cell lines: updated compilation, revised formats and new visualisation tools. *Nucleic Acids Res* **26**: 205–213

Harper JW, Adami GR, Wei N, Keyomarsi K and Elledge SJ (1993) The p21 Cdk-interacting protein Cip1 is a potent inhibitor of G1 cyclin-dependent kinases. *Cell* **75**: 805–816

Harris CC (1993) *p53* at the crossroads of molecular carcinogenesis and risk assesment. *Science* **262**: 1980–1981

Harris CC (1996) p53 tumor suppressor gene: from the basic research laboratory to the clinic – an abridged historical perspective. *Carcinogenesis* **17**: 1187–1198

Hayashi S, Watanabe J and Kawajiri K (1992) High susceptibility to lung cancer analyzed in terms of combined genotypes of P450IA1 and Mu-class glutathione S-transferase genes. *Jpn J Cancer Res* **83**: 866–870

Heim M and Meyer UA (1990) Genotyping of poor metabolisers of debrisoquine by allele-specific PCR amplification. *Lancet* **336**: 529–532

Hirvonen A, Husgafvel-Pursiainen K, Anttila S and Vainio H (1993) The GSTM1 null genotype as a potential risk modifier for squamous cell carcinoma of the lung. *Carcinogenesis* **14**: 1479–1481

Hollstein M, Sidransky D, Vogelstein B and Harris CC (1991) p53 mutations in human cancers. *Science* **253**: 49–53

Hussain SP and Harris CC (1998) Molecular epidemiology of human cancer: contribution of mutation spectra studies of tumor suppressor genes. *Cancer Res* **58**: 4023–4037

IARC Monographs on the Evaluation of Carcinogenic Risks to Humans (1986) Volume 38, *Tobacco Smoking*. IARC: Lyon

Iggo R, Gatter K, Bartek J, Lane D and Harris AL (1990) Increased expression of mutant forms of p53 oncogene in primary lung cancer. *Lancet* **335**: 675–679

Kato S, Bowman ED, Harrington AM, Blomeke B and Shields PG (1995) Human lung carcinogen–DNA adduct levels mediated by genetic polymorphisms in vivo. *J Natl Cancer Inst* **87**: 902–907

Kawajiri K, Eguchi H, Nakachi K, Sekiya T and Yamamoto M (1996) Association of *CYP1A1* germ line polymorphisms with mutations of the *p53* gene in lung cancer. *Cancer Res* **56**: 72–76

Kihara M, Kihara M and Noda K (1994) Lung cancer risk of GSTM1 null genotype is dependent on the extent of tobacco smoke exposure. *Carcinogenesis* **15**: 415–418

Ko LJ and Prives C (1996) p53: puzzle and paradigm. *Genes Dev* **10**: 1054–1072

Kondo K, Tsuzuki H, Sasa M, Sumitomo M, Uyama T and Monden Y (1996) A dose–response relationship between the frequency of p53 mutations and tobacco consumption in lung cancer patients. *J Surg Oncol* **61**: 20–26

Kubbutat MH, Jones SN and Vousden KH (1997) Regulation of p53 stability by Mdm2. *Nature* **387**: 299–303

Kuempel ED, Stayner LT, Attfield MD and Buncher CR (1995) Exposure–response analysis of mortality among coal miners in the United States. *Am J Ind Med* **28**: 167–184

Kunicki PK, Sitkiewicz D, Pawlik A, Bielicka-Sulzyc V, Borowiecka E, Gawronska-Szklarz B, Sterna R, Matsumoto H and Radziwon-Zaleska M (1995) Debrisoquine hydroxylation in a Polish population. *Eur J Clin Pharmacol* **47**:

© 1999 Cancer Research Campaign 

503–505
Legros Y, Lacabanne V, d'Agay MF, Larsen CJ, Pla M and Soussi T (1993) Production of human p53 specific monoclonal antibodies and their use in immunohistochemical studies of tumor cells. *Bull Cancer* **80**: 102–110
Lehman TA, Bennett WP, Metcalf RA, Welsh JA, Ecker J, Modali RV, Ullrich S, Romano JW, Appella E, Testa JR, Gerwin BI and Harris CC (1991) *p53* mutations, *ras* mutations and p53-heat shock protein complexes in human lung cell lines. *Cancer Res* **51**: 4090–4096
Levine AJ (1997) p53, the cellular gatekeeper for growth and division. *Cell* **88**: 323–331
Meijers JM, Swaen GM, Slangen JJ, van Vliet K and Sturmans F (1991) Long-term mortality in miners with coal workers' pneumoconiosis in The Netherlands: a pilot study. *Am J Ind Med* **19**: 43–50
Miller AB (1992) Epidemiology, prevention, prognostic factors and natural history of lung cancer. *Current Opinion Oncol* **4**: 286–291
Miller BG and Jacobsen M (1985) Dust exposure, pneumoconiosis, and mortality of coalminers. *Br J Ind Med* **42**: 723–733
Motykiewicz G, Michalska J, Pendzich J, Perera FP and Chorazy M (1992) A cytogenetic study of men environmentally and occupationally exposed to airborne pollutants. *Mutat Res* **280**: 253–259
Motykiewicz G, Malusecka E, Grzybowska E, Chorazy M, Zhang YJ, Perera FP and Santella RM (1995) Immunohistochemical quantitation of polycyclic aromatic hydrocarbon–DNA adducts in human lymphocytes. *Cancer Res* **55**: 1417–1422
Nakachi K, Imai K, Hayashi S and Kawajiri K (1993) Polymorphisms of the CYP1A1 and glutathione S-transferase genes associated with susceptibility to lung cancer in relation to cigarette smoking dose in a Japanese population. *Cancer Res* **53**: 2994–2999
Perera FP, Hemminki K, Gryzbowska E, Motykiewicz G, Michalska J, Santella RM, Young TL, Dickey C, Brandt-Rauf P, DeVivo I, Blaner W, Tsai WY and Chorazy M (1992) Molecular and genetic damage in humans from environmental pollution in Poland. *Nature* **360**: 256–258
Petersen G (1994) Epidemiology, screening, and prevention of lung cancer. *Curr Opin Oncol* **6**: 156–161
Przygodzki RM, Bennett WP, Guinee MD, Khan MA, Freedman A, Shields PG, Travis WD, Jett JR, Tazelaar H, Pairolero P, Trastek V, Liotta LA, Harris CC and Caporaso NE (1998) p53 mutation spectrum in relation to GSTM1, CYP1A1 and CYP2E1 in surgically treated patients with non-small cell lung cancer. *Pharmacogenetics* (in press)
Rebbeck TR (1997) Molecular epidemiology of the human glutathione S-transferase genotypes GSTM1 and GSTT1 in cancer susceptibility. *Cancer Epidemiol Biomarkers Prev* **6**: 733–743
Ruggeri B, DiRado M, Zhang SY, Bauer B, Goodrow T and Klein-Szanto AJ (1993) Benzo[a]pyrene-induced murine skin tumors exhibit frequent and characteristic G to T mutations in the p53 gene. *Proc Natl Acad Sci USA* **90**: 1013–1017
Ryberg D, Kure E, Lystad S, Skaug V, Stangelend L, Mercy I, Borresen A-L and Haugen A (1994) *p53* mutations in lung tumors: relation to putative susceptibility markers for cancer. *Cancer Res* **54**: 1551–1555
Sambrook J, Fritsch EF and Maniatis T (1989) *Molecular Cloning: a Laboratory Manual*, 2nd edn. Cold Spring Harbor Laboratory Press: Cold Spring Harbor, NY
Shields PG, Bowman ED, Harrington AM, Doan VT and Weston A (1993) Polycyclic aromatic hydrocarbon–DNA adducts in human lung and cancer susceptibility genes. *Cancer Res* **53**: 3486–3492
Speizer FE and Samet JM (1994) Air pollution and lung cancer. In: *Epidemiology of Lung Cancer*, Samet JM (ed), pp. 131–150. Marcel Dekker: New York
Sozzi G, Miozzo M, Donghi R, Pilotti S, Cariani CT, Pastorino U, Della Porta G and Pierotti MA (1992) Deletions of 17p and p53 mutations in preneoplastic lesions of the lung. *Cancer Res* **52**: 6079–6082
Starzynski Z, Marek K, Kujawska A and Szymczak W (1996) Mortality among different occupational groups of workers with pneumoconiosis: results from a register-based cohort study. *Am J Ind Med* **30**: 718–725
Stucker I, Cosme J, Laurent Ph, Cenee S, Beaune Ph, Bignon J, Depierre A, Milleron B and Hemon D (1995) CYP2D6 genotype and lung cancer risk according to histologic type and tobacco exposure. *Carcinogenesis* **16**: 2759–2764
Sugimura H, Caporaso NE, Shaw GL, Modali RV, Gonzalez FJ, Hoover RN, Resau JH, Trump BF, Weston A and Harris CC (1990) Human debrisoquine hydroxylase gene polymorphisms in cancer patients and controls. *Carcinogenesis* **11**: 1527–1530
Takeshima Y, Seyama T, Bennett WP, Akiyama M, Tokuoka S, Inai K, Mabuchi K, Land CE and Harris CC (1993) p53 mutations in lung cancers from non-smoking atomic-bomb survivors. *Lancet* **342**: 1520–1521
Tefre T, Daly AK, Armstrong M, Leathart JBS, Idle JR, Brogger A and Borresen A-L (1994) Genotyping of the CYP2D6 gene in Norwegian lung cancer patients and controls. *Pharmacogenetics* **4**: 47–57
Wang X, Christioni DC, Wincke JK, Fischbein M, Cheng TJ, Mark E, Wain JC and Kelsey KT (1995) Mutations in the p53 gene in lung cancer are associated with cigarette smoking and asbestos exposure. *Cancer Epidemiol Biomarkers Prev* **4**: 543–548
Vahakangas KH, Samet JM, Metcalf RA, Welsh JA, Bennett WP, Lane DP and Harris CC (1992) Mutations of p53 and ras genes in radon-associated lung cancer from uranium miners. *Lancet* **339**: 576–580
Xiong Y, Hannon GJ, Zhang H, Casso D, Kobayashi R and Beach D (1993) p21 is a universal inhibitor of cyclin kinases. *Nature* **366**: 701–704

*© 1999 Cancer Research Campaign*
